# Automated Fabrication of Smart Strain Sensing Threads

**DOI:** 10.3390/mi15101239

**Published:** 2024-10-08

**Authors:** Ege Ozgul, Wenxin Zeng, Sameer Sonkusale

**Affiliations:** Department of Electrical and Computer Engineering, Tufts University, Medford, MA 02155, USA; ege.ozgul@tufts.edu (E.O.); wenxin.zeng@tufts.edu (W.Z.)

**Keywords:** automation, strain sensor, thread sensor, human body monitoring

## Abstract

With favorable properties of stretchability, stitchability, and potential to be woven into a fabric, thread-based sensors have gained considerable interest for wearable devices for smart and connected health applications. To facilitate wearable applications, an easy and reliable way to fabricate these thread-based sensors with good performance and consistency is the key while manufacturing these smart threads. In this paper, we propose an automated thread-coating system that can fabricate thread-based strain sensors with controlled parameters. The platform uses integrated sensors for controlled manufacturing of the threads in a highly compact structure that consists of an innovative tension sensor and a closed-loop thermal management system. Using this new system, a sample thread with a gauge factor of 1.47 and tension sensitivity of 32.64 KΩ/N is prepared. Compared with hand-coated thread, the machine-fabricated thread shows much better sensitivity and consistency. The prepared strain sensor is made into a respiration sensor patch and a limb motion patch to demonstrate its application.

## 1. Introduction

With the increasing demand for wearable sensors, significant research effort has been made for the development and fabrication of strain sensors with low-cost and simple processes. Unlike conventional sensors that consist of rigid and fragile piezoresistors, wearable devices require flexibility and stretchability to be practical in actual applications [1]. As a result, sensors in various formats, such as textiles, nanofiber, or hydrogel, have been studied to monitor the motion of the human body [2,3,4,5,6,7,8]. Having a one-dimensional geometry, the thread is a suitable material to achieve strain sensing with low cost, simple structure, and easy fabrication [9]. Moreover, the intrinsic properties of the thread enable it to be woven onto fabric easily, greatly broadening the capabilities of a wearable device [10].

Thread-based strain sensors have been intensively studied for a wide range of applications [11,12,13,14,15,16,17,18,19,20,21]. In previous projects, thread-based strain sensors have been demonstrated to detect the weight distribution of foot [22], head motion [23], and physical touch [24]. These proposed wearable devices were built using stretchable strain-sensing threads that are lightweight, easy to fabricate, and sensitive. The thread strain sensor was made by manually coating carbon ink on the surface of elastic threads [25]. The process results in conductive carbon particles percolating on the surface of the thread. When stretched, the distance between particles increases. As a result, the resistance of the thread increases. Once the thread is relaxed, the particles move back to the original position and the resistance reverts back. The initial resistivity and sensitivity of the strain-sensing thread are determined by the volume of the carbon ink coated on the thread. However, the manual coating process introduced large variations in the fabricated threads. To achieve consistent carbon-coated thread fabrication, a reel-to-reel thread manufacturing system was designed [22]. The system was able to produce strain-sensing thread with a fixed resistivity, but one could not control the coating thickness, nor monitor the resistivity during the fabrication process.

In this work, we propose an improved strain-sensing thread fabrication system that can achieve more control of the fabrication parameters of the threads. Thread fabrication, monitoring, and testing are all integrated into one system. The platform consists of an innovative strain sensor and a closed-loop thermal management system to ensure consistent coating. The threads fabricated in one run are much more consistent than the hand-coated counterpart. The coating speed is digitally adjustable via the user interface. The maximum speed is limited by the driver’s switching capability and the ink’s viscosity. More viscous ink requires slower coating speeds, while less viscous ink allows for higher speeds. Users can also set lower speeds for improved quality or specific requirements. In this study, the threads are coated at a speed of 12 mm/s. The gauge factor of the fabricated sample thread is 1.47, with a tension sensitivity of 32.64 KΩ/N. The linear regression of the measured data shows an R-squared value of 0.9595. To demonstrate the application of the strain-sensing thread, a respiration sensor and limb motion sensor are shown. The respiration sensor monitors the chest expansion and contraction caused by breathing. The limb motion sensor can monitor the range of motion of arms and legs.

## 2. Design

**Figure 1:**
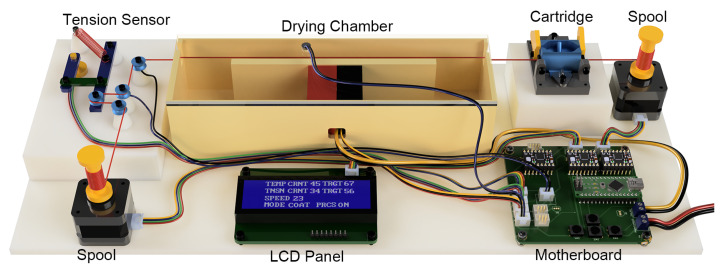
Schematic of the smart thread coating factory.

The smart thread coating system is composed of multiple components that work together to allow the user to manufacture consistent high-quality threads with desired parameters. The conceptual design of the whole system is illustrated in Figure 1. Herein, each of these parts is explained in more depth, including the ink cartridge, the drying chamber, the strain regulator, the microcontroller, and the user interface.

### 2.1. Ink Cartridge

**Figure 2:**
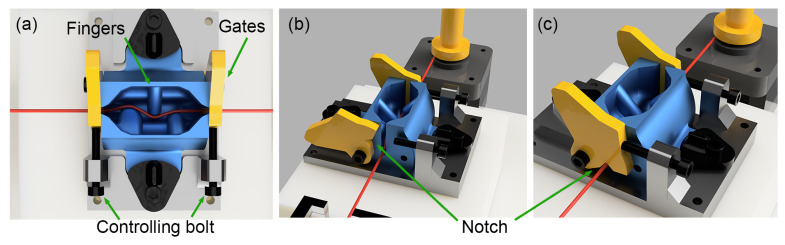
Cartridge (**a**) top view, (**b**) side view with gate open, and (**c**) side view with gate closed.

Instead of directly applying ink to the thread, we propose to pull the thread (Gutermann, Elastic Thread 11 Yards-White, Germany) through custom-designed ink cartridges. The cartridge is a crucial element responsible for the application of conductive ink onto the thread. Our novel design features two gates on either side and three finger-like structures within the cartridge, as illustrated in yellow in Figure 2a. The finger-like structures exert opposing pressure on the thread when it is fed through the cartridge, enhancing the absorption of the carbon ink on the thread. Initially, the gates are kept open to facilitate the installation of the thread, denoted in red, as shown in Figure 2b. Subsequently, the gates are sealed, encapsulating the cartridge, as in Figure 2c, followed by the injection of conductive ink from the top via a syringe. The yellow gates on the cartridge are equipped with V-shaped notches that come in contact with the thread, controlling the residual ink as the thread exits the cartridge. The user can manipulate the notch’s position by adjusting the bolts on the cartridge’s side, thereby altering the gate’s position when closed. Tightening the bolts increases the notch’s size, allowing for a greater quantity of ink to remain on the thread as it departs from the cartridge. Conversely, loosening the thread narrows the gates and reduces the notch’s size, resulting in a diminished ink volume on the thread.

Upon commencement of the coating procedure, a stepper motor draws the thread in and out of the cartridge. Over time, dry ink will accumulate in the cartridge and eventually prevent the cartridge from working. To address this issue, the cartridge is designed for single use, and is detachable from the system, allowing for effortless removal and replacement. It can be 3D printed using PLA, making it highly convenient to fabricate and replace, which is more practical and economical than cleaning a used one.

The thickness of the coating is controlled by two parameters: the viscosity of the ink and the notch size of the cartridge. The notch size can be finely tuned by adjusting a controlling bolt attached to the cartridge. The viscosity of the ink can be adjusted by mixing a thinner solvent into the ink. The carbon-based conductive ink (MG Chemicals, 838AR Carbon Conductive Paint, Canada) with a viscosity of 46 cP at 25 °C. The viscosity can be further adjusted by adding a thinner solvent, allowing precise control over the coating thickness. These parameters enable precise control of the ink application process to ensure uniform coating thickness and quality.

### 2.2. Drying Chamber

**Figure 3:**
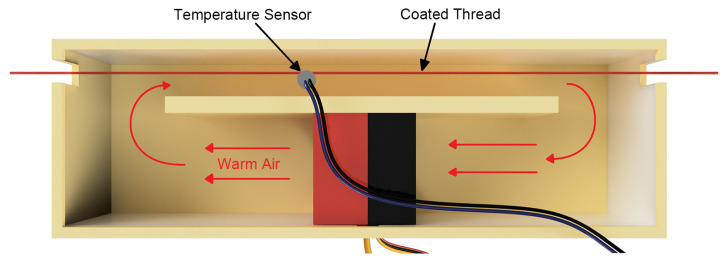
Top view of the schematic of the drying chamber.

Following the ink cartridge, a drying chamber is designed to facilitate the ink’s drying process. As depicted in Figure 3, the chamber allows the thread to move through and directs hot air onto the coated thread. Within this heated enclosure, a fan and a resistive heater work in tandem to circulate the warm air. This enclosed design ensures that the heated air remains confined, allowing the resistive heater to maintain the temperature of the same air without expending additional power. Additionally, a temperature sensor (TIEXYE, 100K NTC 3950 Thermistors, China) is positioned close to the path of the thread. A microcontroller continuously monitors the air temperature in the chamber. The resistive heater is activated when the air temperature is below the desired temperature, and is deactivated when the air temperature is above the target temperature. The fan operates non-stop, ensuring a constant airflow, which allows the heater to immediately change the air temperature.

### 2.3. Thread Tension Regulator

The system utilizes two stepper motors to transfer the raw thread from the input spool to the output spool, as shown in the top-right and bottom-left corners in Figure 1, by rotating the spools independently. During the coating process, a consistent tension of the thread is key to producing a uniform thread that has an identical tension vs. resistance correlation along the thread. A thread tension regulator is designed to monitor and adjust the tension of the thread in real time.

**Figure 4:**
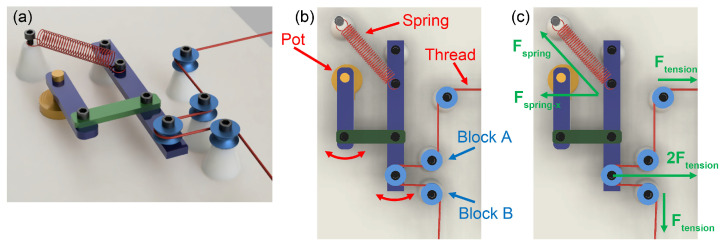
(**a**) 3D model of the tension sensor setup, (**b**) top view of the tension sensor (Pot stands for potentiometer), (**c**) free body diagram.

The tension regulation system consists of a tension sensor and a closed-loop feedback controller. The sensor uses a spring to apply force to the string as shown in Figure 4a,b. When the tension in the string changes, the spring’s length adjusts accordingly, and causes the blue level to pivot to the corresponding degree. A potentiometer is attached at the pivot of the blue level, and is denoted as Pot in Figure 4b. When the thread is at high tension, the blue lever rotates counterclockwise; when there is less tension, it rotates clockwise. The potentiometer correlates with the position of the blue level and senses the spring’s stretching length and the corresponding tension. The feedback controller uses this information to make real-time adjustments to the speed of the start and finish step motors, ensuring that the string’s tension remains constant and precise. A free body diagram of the system is shown in Figure 4c.

**Figure 5:**
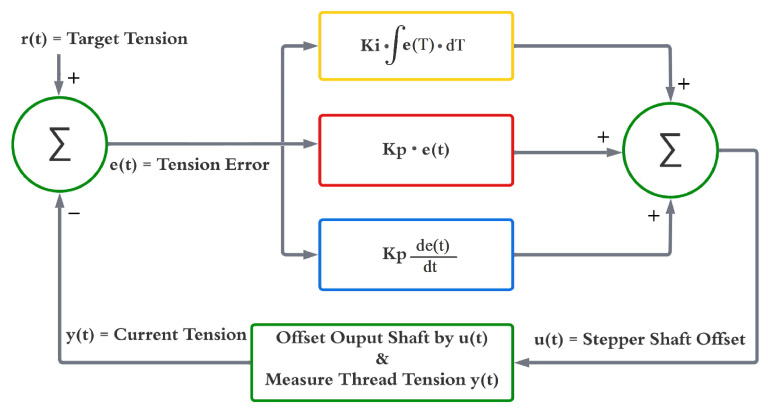
Feedback control algorithm.

A PID feedback controller monitors and regulates the thread tension and keeps it at the target value. The target tension value can be set using the user interface on the device. The PID algorithm shown in Figure 5 is implemented in an Atmega328 microcontroller (Arduino, Nano, Italy), which constantly measures the tension on the thread and adjusts the speed of the output spool based on the error between the current tension and the target. More information about the custom tension sensor is discussed in the following subsection. The PID controller is highly responsive at 12 mm/s coating speed, providing immediate dampening and regulation of the thread. The damping duration is negligible at this coating speed, ensuring smooth operation.

The following PID model is implemented in the firmware of the device. Variable u(t) represents the amount of rotation that the stepper motor covers at the current system loop.

(1) $$u\left(t\right)=k_{p}e\left(t\right)+k_{i}\int e(t)dt+k_{d}\frac{de(t)}{dt}$$

where e(t) is the tension error variable, which equals the target tension minus current tension. $k_{d}$, $k_{i}$, and $k_{p}$ are the PID constants that are used in the feedback system. The values are computed experimentally, and provided below.

(2) $$k_{d}=2.651\times 10^{-7}$$

(3) $$k_{i}=1.238\times 10^{-4}$$

(4) $$k_{p}=1.519\times 10^{2}$$

**Figure 6:**
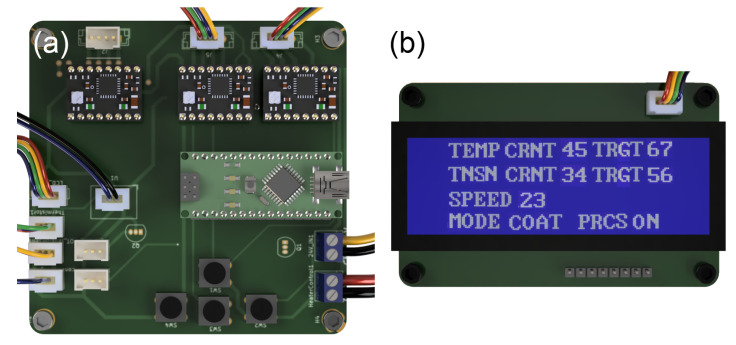
(**a**) Motherboard, (**b**) LCD Panel.

The user interface shown in Figure 6 consists of an LCD screen and four push buttons that are placed on the motherboard. The LCD screen displays the parameters listed below.

- Target temperature and current measured temperature;
- Target thread tension and Current thread tension;
- Spool speed;
- Process state: on, off;
- Process mode: Coat, Consistency Test, Stretch Test.

**Figure 7:**
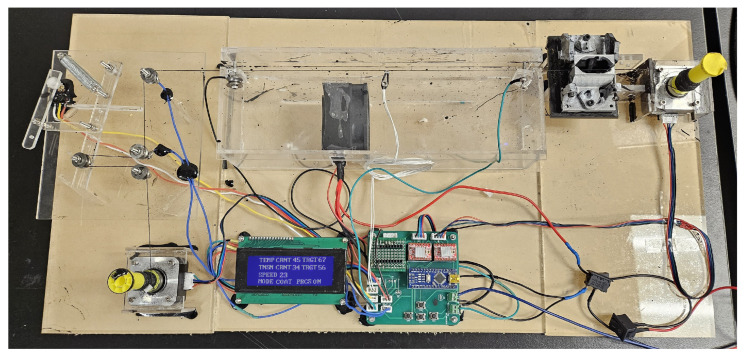
Photo of the entire thread-coating platform.

The four push buttons placed on the motherboard allow the user to adjust the coating parameters and operating mode in the list above. There are three process modes. “Coat” mode is the main coating process, enabling users to define the parametric coating of threads at variable speeds, temperatures, tension levels, and ink thicknesses. It allows users to specify these parameters to create custom threads tailored for specific applications. The “Consistency Test” mode is used for the conductivity test of the entire coated thread. By constantly spinning the input and output spools while regulating the tension, the system can measure the resistance between the two metal blocks connected to the microcontroller, denoted as blocks A and B in Figure 4b. “Stretch test” precisely stretches and releases the coated thread in cycles, which is used for characterizing the coated threads. The consistency and stretch testing modes are utilized to automate the data collection that is presented in the following part. A photo of the whole coating system is shown in Figure 7.

## 3. Thread Analysis and Characterization

**Figure 8:**
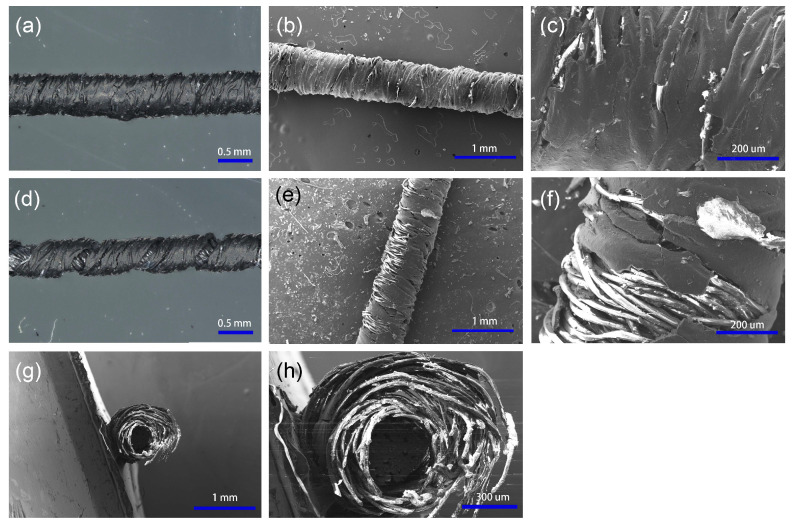
Strain sensor at relaxed state under (**a**) optical microscope and (**b**,**c**) SEM. Strain sensor at stretched state under (**d**) optical microscope and (**e**,**f**) SEM. (**g**,**h**) SEM image of the cross-section sample.

To characterize the fabricated thread, it is analyzed under an optical microscope and SEM, and then tested for its resistance and consistency at different conditions. Figure 8a shows the coated thread in its natural state under the optical microscope. In this case, the carbon particles are tightly connected together; therefore, the electrical resistance is relatively lower (112 KΩ/cm). Figure 8b,c also show the SEM images of the thread in the natural state. It can be seen that multiple carbon particles overlap with each other to create a continuous connection. Figure 8d shows the stretched thread from the optical microscope. In this case, the carbon particles are separated due to the elongation of the thread. As a result, the carbon coating has a higher resistance (251 KΩ/cm). Figure 8e,f show the SEM images of a highly stretched. At this state, some areas show fissures in the coating, and the thread area without carbon covering is visible. The SEM images of the coated thread cross-section are shown in Figure 8g,h.

The coating device is capable of performing tests on the coated threads by using the existing stepper motors and sensors. There are two test processes programmed into the device’s microcontroller: the Consistency Test and Stretch Test. For comparison, a hand-coated thread is tested as well. The hand-coated thread is fabricated by manually applying carbon ink onto the elastic thread. The coated thread is then hung to dry at room temperature with no external tension applied.

### 3.1. Characterization of the Coated Threads

For characterizing the threads, the “Stretch Test” process is selected on the user interface and executed. During the process, only the stepper motor for the output spool rotates to stretch and release the coated thread. For this test, the microcontroller is programmed to stretch and release the thread for a total of 1000 times, while recording the tension and the resistance of the thread. The collected data are continuously sent to a computer via a serial interface.

**Figure 9:**
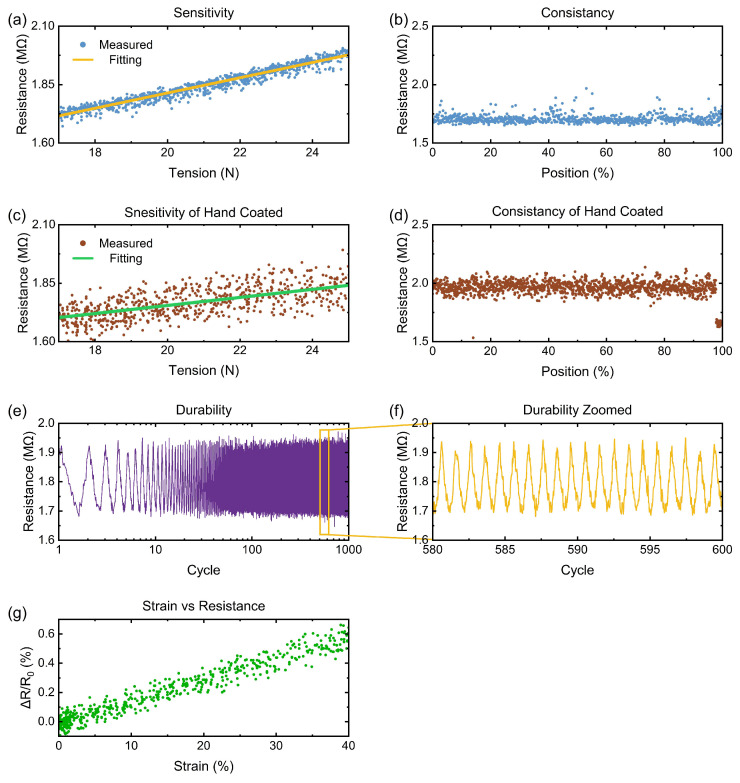
(**a**) Measured and linear fitted tension sensitivity curve of the fabricated strain sensor. (**b**) Resistance consistency along the fabricated thread. (**c**) Tension sensitivity and (**d**) consistency test of hand-coated thread. (**e**) Durability test over 1000 cycles at 40% strain and (**f**) zoomed-in plot. (**g**) Strain vs. resistance plot.

Figure 9a shows the measured and linear regression data between the tension and the resistance of the sample thread that is coated automatically by the system. The tested sample thread is 6 cm long, and has a tension sensitivity of 32.64 KΩ/N. The R-square value of the fitting is 0.9595. Figure 9c displays the collected and linear regression data of the thread that is coated by hand. For the hand-coated thread, the tension sensitivity is 17.24 KΩ/N. The R-squared value of the fitting is 0.5494. The machine-coated thread has much higher tension sensitivity, and is more consistent during the stretching. The durability of the thread has been tested by stretching for 1000 cycles at the maximum strain of 40%. Resistance vs. cycle graphs are plotted in Figure 9e,f. The minimum and maximum resistance values at each cycle stay consistent for 1000 cycles, which indicates the reliable durability of the fabricated thread. The strain vs. resistance curve of the machine-coated sensor is plotted in Figure 9g. With in 40% strain, the gauge factor is 1.47.

### 3.2. Resistance Consistency Test

The coating device is also capable of performing resistance consistency tests. For this test, the device transfers all the coated threads from the input spool to the output spool by using the stepper motors. While moving the thread, the resistance between the metal blocks is measured continuously. These two metal blocks are connected to the ADC of the microcontroller, which measures the resistance between the blocks and sends the data to a computer using the serial interface. Figure 9b shows the consistency data of the thread that is coated automatically by the device, and Figure 9d shows the data of a hand-coated thread. The position of each data point is represented as a percentage of the tested point in the total length of the whole fabricated spool. It can be seen that the machine-coated thread is much more consistent and precise compared to the hand-coated one.

### 3.3. Results on Fabricated Thread

**Figure 10:**
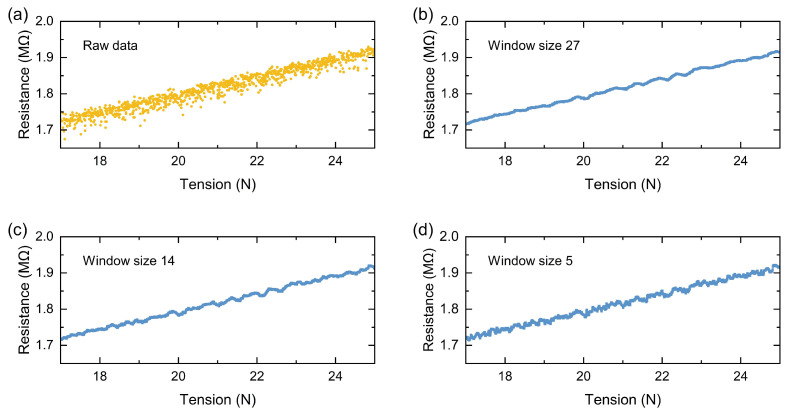
(**a**) Raw data of tension sensitivity of fabricated threads, and tension-resistance relationship after applying a moving average window of (**b**) 27, (**c**) 14, and (**d**) 5 samples.

Reel-to-reel automated fabrication was employed to generate a strain sensing thread and measurements were performed. Strain sensor output will exhibit some variability in resistance due to the stochastic nature of carbon particles forming a resistive network for a given stretch. One is expected to employ a smoothing algorithm for reliable measurements. A moving average filter with window sizes of 5, 14, and 27 sampled points was employed to smoothen the resistance output. As shown in Figure 10, with a larger window size, the moving average reduces the noise significantly at the expense of slower readout. More advanced filtering algorithms can be utilized depending on the complexity of the application and the processing power available on the system, which is not the focus of this paper.

## 4. Application

**Figure 11:**
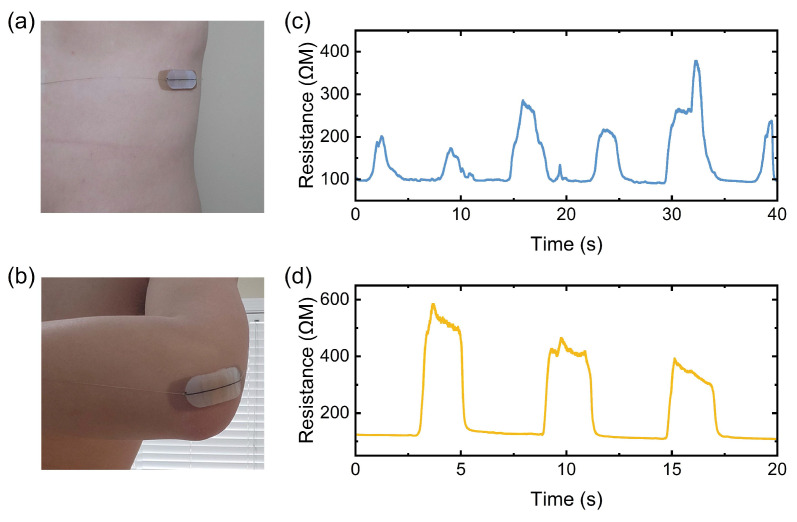
Images of (**a**) reparation sensing patch and (**b**) limb motion patch. Real-time resistance data of (**c**) respiration and (**d**) elbow motion.

To demonstrate the application of the fabricated strain-sensing thread, a wearable sensor patch was designed. The sensor patch can be applied to the human body to record respiration or limb movement, depending on the placement location [22,23]. The strain-sensing thread is connected to a resistance trans-impedance amplifier (TIA) to characterize the resistance in real-time, followed by a 10-bit ADC and a microcontroller, which transmits the data wirelessly to a terminal. Figure 11a,b show the sensor patch applied to the chest and elbow of a person, respectively. A piece of the strain-sensing thread was placed on a regular band-aid. Eco-flex was used to fix the thread in place with the band-aid as well as protect the thread from scratching. Conductive silver strings were used to connect the thread to the TIA. The length of the strain-sensing thread was chosen so that its initial resistance is 100 MΩ.

For respiration monitoring, the band-aid was applied to the chest when the person exhaled. As the person inhales, his chest expands, thus stretching the thread and resulting in an increase in the resistance. When the person exhales, the chest volume shrinks, allowing the thread to retract and impedance drops back to 100 MΩ. Figure 11c shows the measured respiration data in real-time. The overall measured respiration rate is around nine times per minute. Moreover, as the person performs deep breaths and shallow breaths irregularly, the magnitude of the resistance responses accordingly and accurately captures the different intensities and duration of respiration. Future studies may help correlate the results of respiration sensors to actual lung function.

To monitor limb movement, the sensor patch was applied to the elbow. The sensor patch stretches when the arm is bent. Figure 11d shows the measured resistance for elbow flexing. At the maximum of $150^{\circ }$ elbow flexing, the resistance of the thread increased from 100 MΩ to around 550 MΩ. As the elbow was relaxed, the resistance immediately decreased back to 100 MΩ. When the elbow was only flexed $90^{\circ }$, the resistance increased to around 420 MΩ. At a lower $45^{\circ }$ flexing, the resistance only increased to 350 MΩ. By mapping the resistance of the sensor patch to the elbow flexing position, one can monitor the motion of the forearm, as well as other body joints. Future efforts can be performed to correlate individual limb motion to the strength and duration of physical activity. The two combined examples of respiration and limb movement show promise of smart strain sensing threads for IoB applications. This is only made possible due to the high quality of the automated coating process.

## 5. Conclusions

In this work, an automatic strain-sensing thread-coating system with a dimension of 70 by 35 cm was presented. The system is capable of continuously coating carbon ink onto elastic threads with controlled parameters. The thread in this work is coated at 12 mm/s. The fabricated thread has a linear strain-to-resistance ratio. The gauge factor and tension sensitivity are 1.47 and 32.64 KΩ/N, respectively, and remain unchanged after 1000 stretching cycles. Compared to the hand-coated thread, the system provides much higher consistency. To demonstrate the application of the fabricated thread, a wearable patch was designed to monitor respiration and limb motion. The strain-sensing thread converts the physical length change on the surface of the human body to resistance. An integrated wireless platform enabled remote access and processing. This compact thread-coating platform could also be extended for coating other functional inks such as silver and silver chloride, or carbon nanotubes for making electrochemical sensors. It can also be used to coat semiconducting inks to make photovoltaic cells or transistors and will be the focus of future work.

## Data Availability

The 3D-printing file for the ink cartridge and the electronics file can be found at https://github.com/egeozgul/Smart-Sensing-Thread-Factory/tree/main (github.com), accessed on 1 September 2024. Other data are contained within the article.
